# Polyvinyl Alcohol Hydrogel Irradiated and Acetalized for Osteochondral Defect Repair: Mechanical, Chemical, and Histological Evaluation after Implantation in Rat Knees

**DOI:** 10.1155/2012/582685

**Published:** 2012-11-01

**Authors:** N. A. Batista, A. A. Rodrigues, V. P. Bavaresco, J. R. L. Mariolani, W. D. Belangero

**Affiliations:** ^1^Orthopaedic Biomaterials Laboratory, Faculty of Medical Sciences, University of Campinas (UNICAMP), Rua Tessália Vieira de Camargo, no. 126, Cidade Universitária “Zeferino Vaz”, 13083-887 Campinas, SP, Brazil; ^2^Department of Plastic, Technical College Campinas (COTUCA), University of Campinas (UNICAMP), 13083-887 Campinas, SP, Brazil

## Abstract

Polyvinyl Alcohol (PVA) hydrogel plugs were implanted in artificial osteochondral defects on the trochlear groove of rat knees. After 0, 3, 6, 12, and 24 weeks of followup, samples containing the implants were mechanically evaluated by creep indentation test, chemically, and histologically by optical microscopy. The mechanical test pointed towards an increase of the implant creep modulus and the chemical analysis exhibited an increasing concentration of calcium and phosphorus within the implants over time. Optical microscopy showed no foreign body reaction and revealed formation, differentiation, and maintenance of new tissue at the defect/implant interface. The absence of implant wear indicated that the natural articular lubrication process was not disturbed by the implant. The performance of the irradiated and acetalized PVA was considered satisfactory for the proposed application.

## 1. Introduction

The articular cartilage consists of a high-specialized, low friction tissue that covers the epiphyses with the function of enabling bones do glide over each other and to absorb impacts within the joint without causing wear; this cartilage has a poor capacity of renewal [1–3]. Its viscoelastic behavior depends on the intrinsic mechanical properties of the extracellular matrix, the presence of collagen gel in the matrix, and the flow of the interstitial water due to the load applied during the movement [4, 5].

The articular cartilage can suffer degeneration, beginning with a degradation of the cartilage itself (chondral lesion) that may reach and expose the subchondral bone (osteochondral lesion) [6]. The damages can be of traumatic origin, such as articular fractures, ligament, or meniscus lesions, or of inflammatory origin, as in autoimmune or metabolic diseases [7]. The Articular Cartilage exhibits a low-intrinsic ability of self-repair, this is due to a lack of vascularization and due to the low-metabolic activity of the mature chondrocytes, which in turn, limit the supply of growth factors, responsible for the cellular differentiation and proliferation [2]. Furthermore, the lack of innervation delays the manifestation of clinical symptoms, facilitating a quiet advance of the articular degeneration [8].

Nowadays, treatment options are based on abrasion, microfractures, mosaicplasty, first- and second-generation autologous chondrocyte implantation, and allogeneic osteochondral grafting [9–16]. These techniques have drawbacks such as morbidity of the donor area, reduced mechanical stability of the new tissue compared with the normal cartilage, genetic incompatibility between donor and recipient and high cost, among other problems, and none of these have so far proven to be effective enough to ensure complete long-term regeneration [17].

In view of this, the development of osteochondral implants that mimic the physical, chemical, and mechanical properties of the articular cartilage has been proposed [4, 17, 18]. Such implants for osteochondral defects consist of scaffolds that must allow simultaneous growth of both cartilage tissue and subchondral bone. They may neither be cytotoxic nor elicit inflammatory response of the host tissue; otherwise they will not allow cartilage regeneration and will be encapsulated by fibrous tissue.

Among the materials used for osteochondral defect repair, bioceramics, such as bioglass, hydroxyapatite, and calcium phosphate, occupy a prominent place [18]. Noteworthy is also the use of synthetic biodegradable polymers such as poly(*α*-hydroxy ester), poly(ethylene glycol) (PEG), and poly(caprolactone) (PCL) [19]. These polymers exhibit the advantages of easy processing, low-processing costs, controlled biodegradability, and FDA-approval [20]. Amongst them, the polymeric hydrogels have proven to be a promising class of materials for osteochondral repair, since their physical and chemical characteristics can resemble that of the articular cartilage and the subchondral bone [4, 21–26]. Polyvinyl alcohol (PVA) crosslinked by electron beam irradiation deserves special attention thanks to its chemical stability against biological fluids, good elasticity, adequate mechanical strength, hydrophilicity, physical properties similar to those of the cartilage, and low processing costs [27–30].

Preliminary *in vitro* and *in vivo *tests of PVA hydrogel have exhibited promising results [31, 32], qualifying PVA for further tests. Thus, the aim of this study was to evaluate the *in vivo* performance of PVA cross-linked by electron beam irradiation implanted in Wistar rats' knees by means of mechanical, chemical, and histological tests.

## 2. Materials and Methods

### 2.1. Preparation and Characterization of Polyvinyl Alcohol (PVA)

**Figure 1:**
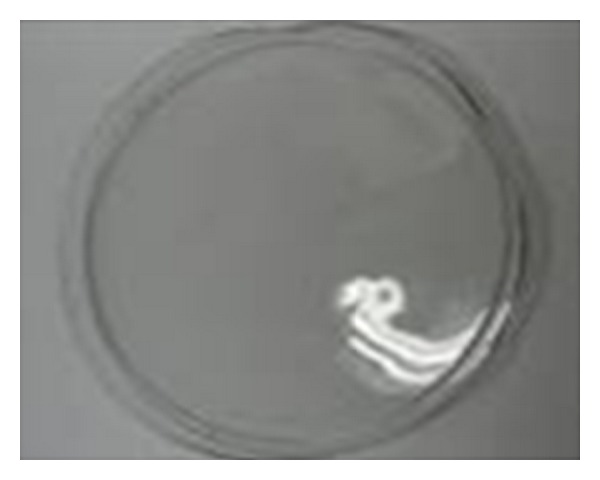
PVA membrane.

Aqueous solution of PVA was obtained by 10% Sigma-Aldrich mW 89,000–98,000 g/mol, 99% hydrolyzed and was prepared and homogenized at 70°C for 1 h in magnetic stirrer. The solution was transferred to a Petri dish and kept at room temperature for 7 days resulting in 1 mm thick membranes (Figure 1). The membranes were acetalised by a chemical treatment composed of formaldehyde solution 40% (w/w) (Aldrich), concentrated sulfuric acid 50% (w/w) (Aldrich), and 300 g anhydrous sodium sulfate (Aldrich). The membranes were maintained in this solution under constant stirring at 70°C for 24 h. After being washed and hydrated in running distilled water, the samples were crosslinked by electron beam irradiation at 25 kGy produced by a Radiation Dynamiton electron beam accelerator (Institute of Energy and Nuclear Research, São Paulo, Brazil) [32].

**Figure 2:**
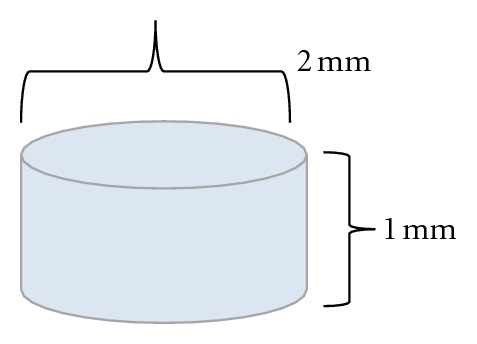
Osteochondral implant PVA.

Thereafter, the samples were hydrated and swelled in 0.9% sodium chloride (NaCl) solution for 48 h and osteochondral implants (plugs 2 mm in diameter and 1 mm in height) were obtained by punched cut outs (Figure 2). The plugs were stored at low temperature in sterile Falcon tubes containing 0.9% NaCl until implantation.

### 2.2. Animal Care and Experimental Groups

Forty-four male rats (~380 g) were anesthetized by intravenous injection of 25 mg/kg pentobarbital according to the ethical protocol approved by the Ethics Committee in Animal Experimentation of the University of Campinas, São Paulo, Brazil (protocol n° 1047-1/2008). The knee joint was accessed by a medial parapatellar incision and a twist drill fitted with a depth stop was used to produce cylindrical osteochondral defects (2 mm in diameter and 1 mm deep) in the intercondylar region.

The animals were divided into 6 groups: 5 experimental groups with 8 animals each, for implantation of the PVA samples (0, 3, 6, 12, and 24 weeks followup referred to as EG00, EG03, EG06, EG12, and EG24, resp.) and a “cartilage” control group (CG) with 08 animals, whose articular cartilage was kept intact.

#### 2.2.1. Surgical Procedures and Experimental Design

**Figure 3:**
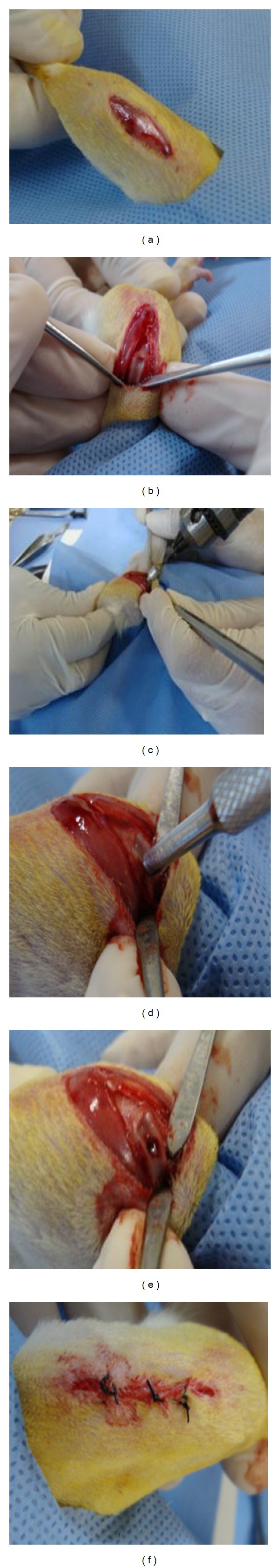
Surgical procedure: (a) skin incision; (b) exposure of the intercondylar region; (c) drilling the osteochondral defect with a 2 mm diameter and 1 mm depth stop; (d) PVA implant; (e) defect filled with the PVA implant; (f) suture.

The hydrogel plugs were inserted under pressure into the defects with the aid of a trephine and sit flush with the surface of the adjacent articular cartilage (Figure 3). The joint capsule and the skin were sutured and the animals were placed in cages where they received Paracetamol solution 25 mg/kg for 24 h. After that, only food and water were offered *ad libitum* until the animals were sacrificed.

### 2.3. Creep Indentation Test (CIT)

Six condyles from each EG and CG group (*n* = 36) were randomly selected and stored in 0.9% NaCl solution until the test, in order to avoid tissue autolysis. The test was carried out on a universal testing machine EMIC DL300 (Curitiba, Brazil). A load of 4.905 N (0.5 kgf) was applied to the implant surface by a hemispherically ended indenter (1.4 mm diameter) and kept for 180 seconds [33] and curves of indenter penetration depth versus time were plotted from test data that were continuously registered by the test machine software (TESC 3.04). The creep modulus for the hydrogels *in situ *at 180 seconds after load application was calculated according to the equation below (originally deduced for sheets of vulcanized rubber and successfully used by Kempson et al. [34] for human articular femoral cartilage):

(1) $$\begin{array}{c} E=\frac{9\times 10^{-6}p}{16\sqrt{r}}{\left[\frac{1-e^{\left(-0.42\;t/a\right)}}{h}\right]}^{3/2},\\ a=\sqrt{\left(2rh-h^{2}\right)}\left[m\right]. \end{array}$$

### 2.4. X-Ray Fluorescence Analysis (EDXRF)

Three hydrogel plugs from each EG (*n* = 12) and three nonimplanted plugs EG00 (*n* = 3) were subjected to XRF analysis, in order to assess the incorporation of calcium (Ca), phosphorus (P), and sulfur (S) by the PVA. After removal, the plugs were dried at room temperature, put into XRF polyethylene sample cups (Chemplex), and analyzed on an energy dispersive X-ray fluorescence spectrometer (Shimadzu EDX 700) with rhodium X-ray tube and semiconductor Si (Li) detector (energy resolution of 165 eV (Mn Ka line), under the following conditions: 3 mm diameter incident beam collimator, X-ray tube voltage of 15 kV, exposure time of 100 s, and detector dead time of 25% [35, 36]. The software AXIL was employed to interpret the results and calculate the concentration of the elements in the samples.

### 2.5. Optical Microscopy (OM)

After the mechanical test, fourteen samples from the EG group (*n* = 40) were fixed in buffered 10% formaldehyde solution (pH 7.5) for 48 h, decalcified in a solution of ethylenediaminetetraacetic acid disodium (EDTA) 7% (w/w), hydrochloric acid 100% 140 mL (w/w), sodium tartarate 0.15% (w/w), sodium, and kaulium tartarate 0.9% (w/w) in distilled water for 15 days, washed in distilled water for removal of residual decalcifying solution, immersed in ethanol 70%, and finally embedded in paraffin. The samples were prepared for OM according to the previously described protocol [23].

Semithin serial sections (4 *μ*m thick) were cut on a rotatory microtome Leica 2155 and stained with hematoxylin eosin (HE). Images were obtained with a Leica DMLB 100 S microscope (magnification 200 *μ*m). The images were analyzed for the absence, moderate, or intensive presence of the following structures: granulation tissue (GT), collagen fibers (CF), mineralized bone matrix (MB), neoformed bone tissue (NB), and collapse of neoformed tissue (CN).

The results obtained by OM were evaluated according to the criteria: absent (+), moderate (++), and (+++) intensive as described in Table 1.

## 3. Results

### 3.1. Creep Indentation Test (CIT)

**Figure 4:**
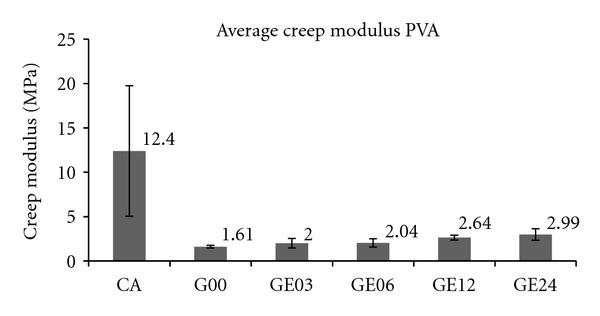
Average creep modulus and respective standard deviations for the rat articular cartilage (CA), control group (G00), and the experimental groups (EGs) according to follow-up time. Confidence level (*P* < 0.05) was obtained through Anova multiple comparison analysis statistics.

Figure 4 shows the average creep modulus and respective standard deviation for each experimental control group.

### 3.2. X-Ray Fluorescence Analysis (EDXRF)

**Figure 5:**
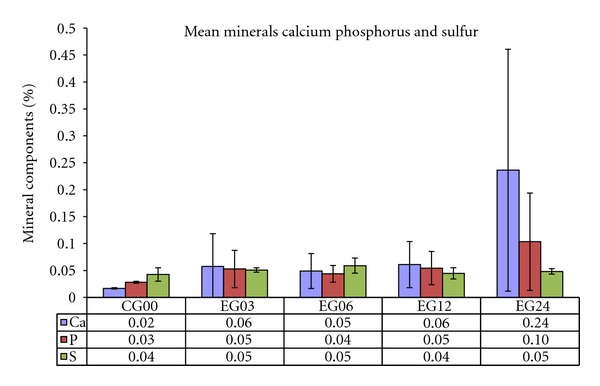
Mean concentrations of calcium, phosphorus, and sulphur found in the implanted plugs according to follow-up time (EGs) and PVA not implanted *in vivo* (GC00). Confidence level (*P* < 0.05) was obtained through Anova multiple comparison analysis statistics.

Figure 5 shows the average concentrations of calcium, phosphorus and sulfur according to follow-up time.

### 3.3. Optical Macroscopy and Microscopy (OM)

**Figure 6:**
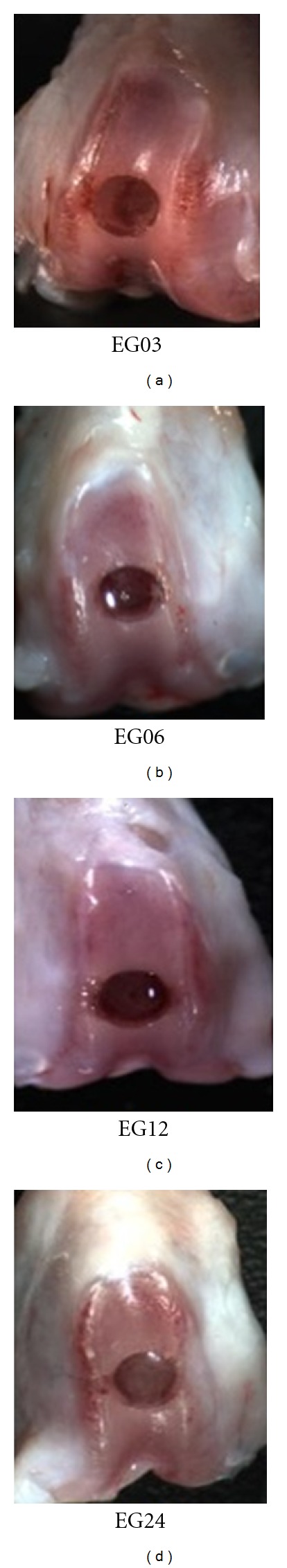
EGs stereoscopic images. Maintenance of PVA aspect and of adjacent cartilage can be observed, furthermore there was an absence of neoformed tissue surrounding the implant and no PVA implant collapse. Magnification 10x.

Figure 6 containing macroscopic images of EGs after animals were sacrificed.

**Figure 7:**
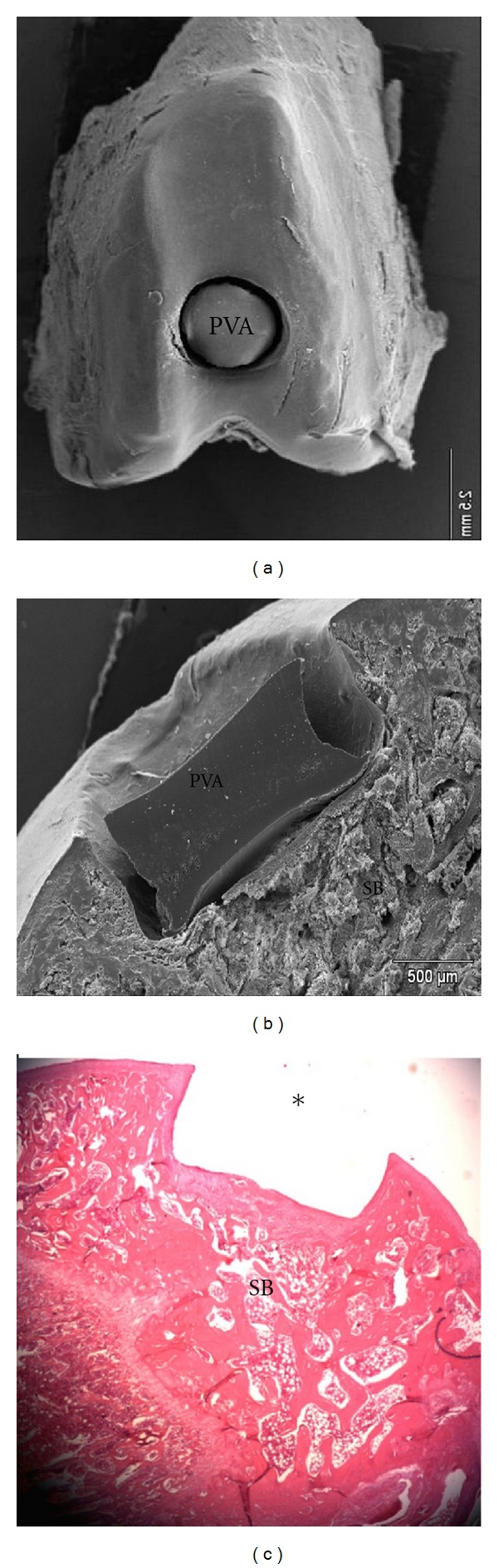
Scanning electron microscopy of the PVA surface in the condyle. (a) Scanning electron microscopy of the PVA interface of the osteochodral defect. (b) Histological section of the condyle-*observe absence of PVA which was removed during the treating process for histological analyses. (c) HE Staining 25x.

According to Mow et al. [4] implants, although inert when implanted, are involved by a fibrous capsule of connective tissue. In this study, this was confirmed by the absence of tissue formation on the PVA, as observed (Figure 7).

**Figure 8:**
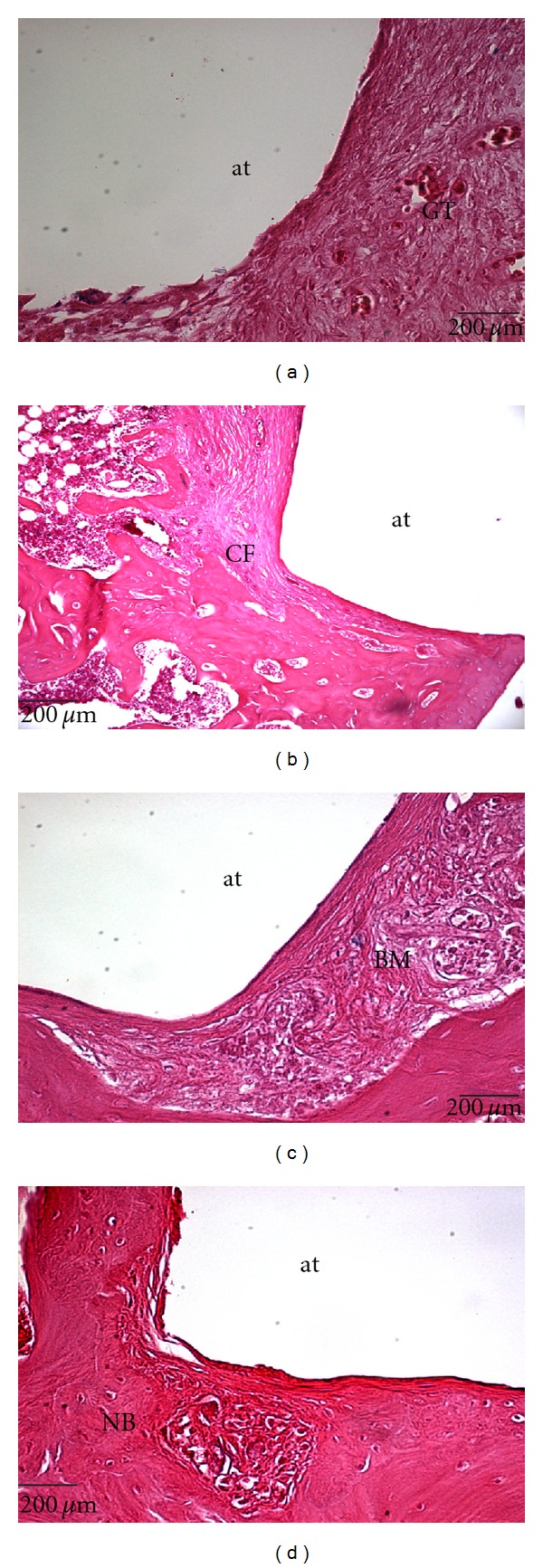
Longitudinal sections of samples containing the PVA implants. (a) EG03-presence of granulation tissue (GT); (b) EG06-presence of collagen fibers (CF); (c) EG12-presence of mineralized bone matrix (BM); (d) EG24-presence of neoformed bone tissue (NB). at = absence of tissue over the implant surface. Stained with Hematoxylin and Eosin.

Figure 8 shows images obtained by OM. As can be noticed, the implant was removed from all samples during the processing for histological analysis. This occurred as there was no tissue growth over the implant surface.

**Figure 9:**
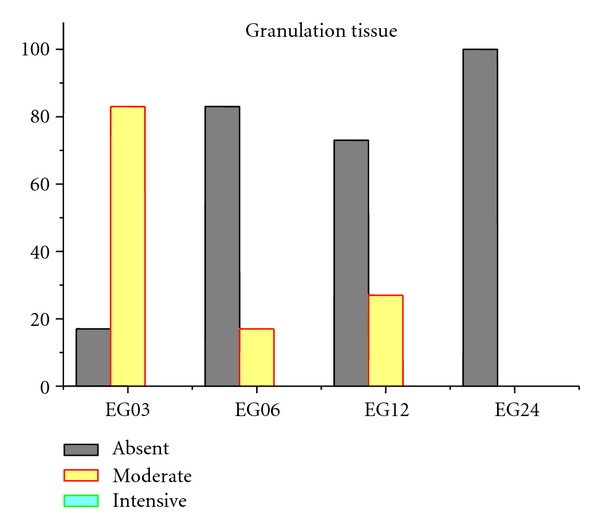
Mean granulation tissue in EGs samples. A 68% absence and 32% moderate presence was observed, however intensive presence was not observed in any of the EGs.

**Figure 10:**
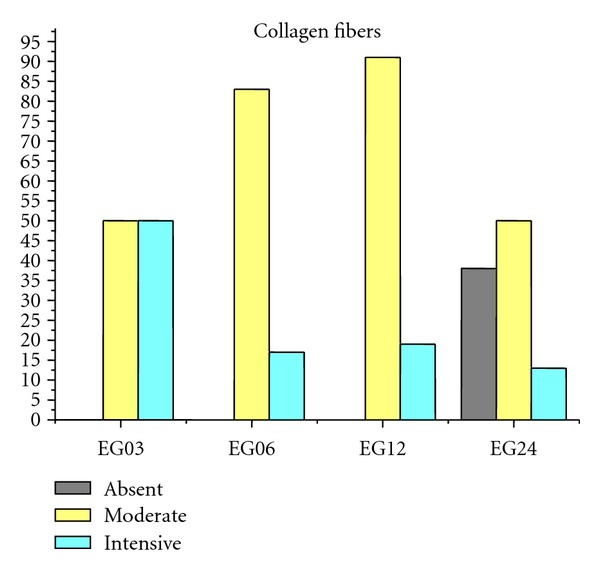
Mean EGs collagen fibers. Absence: 9%, moderate: 69%, and intensive: 22%.

**Figure 11:**
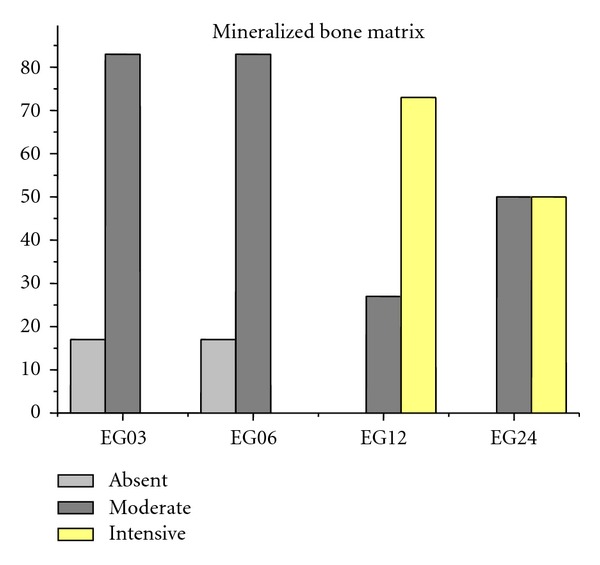
Mineralized bone matrix mean of EGs. Absence: 9%, moderate: 61%, and intensive: 30%.

**Figure 12:**
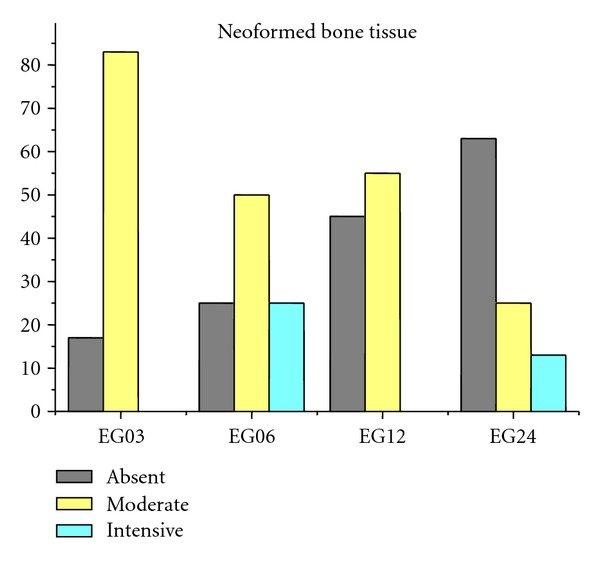
Mean neoformed bone tissue of EGs. Absence: 37%, moderate presence: 53%, and intensive presence: 10%.

**Figure 13:**
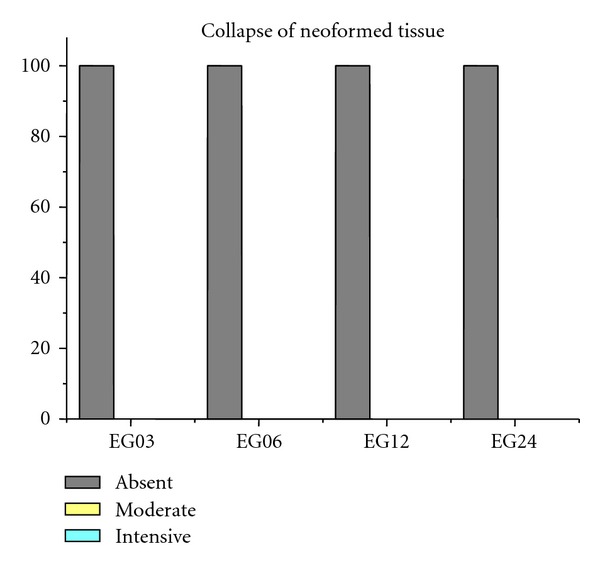
EGs collapse of neoformed tissue was 100% absent in samples.

Figures 9, 10, 11, 12, and 13 present mean scores obtained according to the criteria established in Table 2, these scores were given according to absence, moderate, or intensive presence of tissue on EGs according to duration of followup.

## 4. Discussion

Several studies have shown that the mechanical behaviors of the articular cartilage under *in vitro *and *in vivo* compression cannot be compared with each other. Contrary to *in vitro*, *in vivo* tissue recovers its original dimensions after load removal thanks to a number of factors that define the elasticity and water absorption capacity of its complex structure, such as pressure, viscosity, temperature and macromolecular content of the interstitial fluid, and the concentration and type of dissolved ions [1, 3, 4].

Figure 4 clearly shows that the average creep modulus of the implanted hydrogels increased over time. The increase can be partially attributed to the regrowth of the subchondral bone at the defective bottom (Figures 6 and 7), which provided a stiffer bed for the implant over time, thus restricting deformation and resulting in a higher calculated creep modulus.

Comparing the creep modulus obtained in this study for rat cartilage (Figure 4) and that obtained by Kempson et al. [34] for human femoral cartilage (8.4–10.6 MPa), the values obtained for the PVA hydrogel, though relatively low, were still within the same order of magnitude.

Another contribution to the increase of the creep modulus over time could be assigned to the absorption of calcium and phosphorus by the implant, evidenced by the results of the EDXRF analysis (Figure 5).

The EDRFX analysis (Figure 5) revealed increasing concentrations of Ca and P in the implants over time, probably due to absorption of calcium phosphate, and a steady concentration of S in comparison to the GC00 (not implanted). The consequences of the absorption of calcium phosphate are still unclear and deserve further investigations. It is well known that all implants, although inert when implanted, are involved by fibrous capsule of connective tissue [4].

EDRFX results indicated an increase in Ca and P levels over follow-up time. Further studies are being carried out to determine if the analyzed elements were detected in or on the surface the PVA.

According to the macroscopic results of the EGs (Figure 6), the integrity of the surface of PVA and adjacent CA were maintained by the normal lubrication process.

As observed in the macroscopic images (Figures 7(a) and 7(b)), the PVA implant kept its integrity, with no sign of wear, in all EGs and in the adjacent CA. The result indicates that the process of synovial fluid lubrication was maintained during the entire followup.

From the OM (Figure 8), the quality of the interface between implant and the surrounding tissue (subchondral bone) was satisfactory, with no foreign body reaction. The PVA hydrogel could bear the load without rupture or wear and offered a suitable and stable environment for the formation of new bone at the interface with the host tissue.

The implant surface appears to have maintained its integrity due to the presence of synovial fluid. That was possible because the electron beam crosslinking of the PVA promoted the formation of a polymeric structure that was strong, yet still complacent and capable of absorbing and retaining synovial fluid, which kept low the friction between the articular surfaces and counter surfaces in all EGs.

The absence of signs of implant rejection in almost all samples attests the good compatibility between the hydrogel and the tissues surrounding the artificial osteochondral defect (Figures 9–13).

## 5. Conclusion

The PVA hydrogel implant bore the load without wear or rupture, offered a stable and suitable environment for growing new bone at the interface with the host tissue, and did not disturb the natural articular lubrication. The results of these mechanical, chemical, and histological analyses indicate PVA hydrogel irradiated and acetalised as a promising biomaterial for repairing osteochondral defects.

## Figures and Tables

**Table 1:** Criteria and score evaluation by GE's optical microscopy.

Evaluated criteria	Scores
Granulation tissue (GT)	Absent (+) Moderate (++) Intensive (+++)
Collagen fibers (CF)	
Mineralized bone matrix (MB)	
Neoformed bone tissue (NB)	
Collapse of neoformed tissue (CN)	

**Table 2:** Percentage of the results according to criteria and attributed the scores.

Scores criteria groups (EGs)				
absent (+) moderate (++) intensive (+++)				
Evaluation criteria	Intensity of the presence (%)			
	EG03	EG06	EG12	EG24

Granulation tissue (GT)	83 (+)	83 (+)	73 (+)	100 (+)
	17 (++)	17 (++)	27 (++)	

Collagen fibers (CF)	50 (++) 50 (+++)	83 (+) 17 (++)	91 (++) 9 (+++)	37,5 (+) 50 (++) 12,5 (+++)

Mineralized bone matrix (MB)	17 (+)	17 (+)	27 (++)	50 (++)
	83 (++)	83 (++)	73 (+++)	50 (+++)

Neoformed bone tissue (NB)	17 (+) 83 (++)	25 (+) 50 (++) 25 (+++)	45 (++) 55 (+++)	62,5 (+) 25 (++) 12,5 (+++)

Collapse of neoformed tissue (CN)	100 (+)	100 (+)	100 (+)	100 (+)
